# High‐Density Microporous Li_4_Ti_5_O_12_ Microbars with Superior Rate Performance for Lithium‐Ion Batteries

**DOI:** 10.1002/advs.201600311

**Published:** 2017-01-25

**Authors:** Linkai Tang, Yan‐Bing He, Chao Wang, Shuan Wang, Marnix Wagemaker, Baohua Li, Quan‐Hong Yang, Feiyu Kang

**Affiliations:** ^1^ Engineering Laboratory for the Next Generation Power and Energy Storage BatteriesGraduate School at ShenzhenTsinghua UniversityShenzhen518055P. R. China; ^2^ Laboratory of Advanced MaterialsSchool of Materials Science and EngineeringTsinghua UniversityBeijing100084P. R. China; ^3^ Department of Radiation Science and TechnologyDelft University of TechnologyMekelweg 152629JBDelftThe Netherlands

**Keywords:** high tap densities, Li‐ion batteries, lithium titanate, microporous microbars, NH_4_HCO_3_ templates

## Abstract

Nanosized Li_4_Ti_5_O_12_ (LTO) materials enabling high rate performance suffer from a large specific surface area and low tap density lowering the cycle life and practical energy density. Microsized LTO materials have high density which generally compromises their rate capability. Aiming at combining the favorable nano and micro size properties, a facile method to synthesize LTO microbars with micropores created by ammonium bicarbonate (NH_4_HCO_3_) as a template is presented. The compact LTO microbars are in situ grown by spinel LTO nanocrystals. The as‐prepared LTO microbars have a very small specific surface area (6.11 m^2^ g^−1^) combined with a high ionic conductivity (5.53 × 10^−12^ cm^−2^ s^−1^) and large tap densities (1.20 g cm^−3^), responsible for their exceptionally stable long‐term cyclic performance and superior rate properties. The specific capacity reaches 141.0 and 129.3 mAh g^−1^ at the current rate of 10 and 30 C, respectively. The capacity retention is as high as 94.0% and 83.3% after 500 and 1000 cycles at 10 C. This work demonstrates that, in situ creating micropores in microsized LTO using NH_4_HCO_3_ not only facilitates a high LTO tap density, to enhance the volumetric energy density, but also provides abundant Li‐ion transportation channels enabling high rate performance.

## 1 Introduction

Rechargeable lithium‐ion batteries (LIBs) have been demonstrated to be the most appropriate energy storage devices for large‐scale energy storage owing to their high energy density and long cycle life.^1^, ^2^ As the most widely commercialized anode material, the graphite anode suffers from several disadvantages, for instance, severe irreversible lithium dendrites, poor high rate charge capability, formation of a solid electrolyte interface (SEI), and serious safety issues.^3^, ^4^, ^5^, ^6^ Thus, it cannot satisfy the increasing demand for fast energy storage and large‐scale devices which requires improvement of the battery current densities, cycling stability, safety, and low‐temperature charge properties.

Among the many anode candidates spinel Li_4_Ti_5_O_12_ (LTO) has proven to be a promising alternative anode material.^7^ On the one hand, spinel LTO possesses a high constant Li‐insertion voltage (≈1.55 V vs Li^+^/Li) which suppresses SEI formation and dendritic lithium. On the other hand, the zero‐strain volume expansion upon Li‐ion insertion (≈0.2% volume change), is additionally responsible for its long cycle life and is also expected to be responsible for its fast charging and discharging characteristics.^8^, ^9^, ^10^ In addition, the cubic spinel LTO structure provides a 3D Li‐ion diffusion network facilitating high Li‐ion conductivities.^6^, ^8^

Although LTO suffers from a low intrinsic electronic conductivity (≈10^−13^ S cm^−1^) and poor Li‐ion mobility,^11^, ^12^, ^13^, ^14^ intermediate compositions drastically increase both the electronic conductivity^15^, ^16^, ^17^, ^18^ and Li‐ion mobility^11^, ^12^, ^13^, ^14^ to exploit the intrinsic high rate LTO materials properties requiring excellent electronic and Li‐ion wiring throughout the electrodes. To address this issue, many approaches have been developed, mainly concentrating on adding a conductive coating layer, reducing the particle size, and shortening of the electron and Li‐ion diffusion distances. For instance, metal, carbon, and graphene coating have been shown to increase the surface electronic conductivity of LTO. Nanosized LTO possesses high Li‐ion ionic conductivity, for instance, nanoparticles,^3^, ^19^ nanofibers,^20^, ^21^ nanosheets,^9^, ^22^ nanotubes,^23^ nanowires,^24^ nanoarrays,^23^, ^24^ and nanoscopic porous frameworks.^25^ Unfortunately, as LTO particle size decreases to the nanoscale, the specific surface area of the materials increased greatly, which significantly introduces irreversible reactions with the electrolyte solution.^26^, ^27^ Furthermore, nanostructured materials suffer from a very low tap density leading to a low volumetric energy density.^28^, ^29^ For electrodes with a high tap density, the Li‐ion transport throughout the electrodes has been shown to limit the capacity at high (dis)charge rates.^30^, ^31^ Therefore, the challenge is to prepare electrodes that combine a high tap density with excellent Li‐ion and electron transport, where the presence of interconnected micrometer sized pores appears to be an effective route.^31^, ^32^, ^33^

Microscale LTO materials have high tap density but present unsatisfactory capacity and low rate performance (Figure S1 and Table S1, Supporting Information) because of the relatively low Li^+^ migration rate through the LTO bulk materials.^34^ Monodispersed mesoporous Li_4_Ti_5_O_12_ sub‐microspheres and quasi‐spherical micro‐size Li_4_Ti_5_O_12_ were prepared and their tap densities are as high as 1.62 and 1.38 g m^−3^, respectively.^35^, ^36^ Whereas, above materials present obviously lower rate performance. Mesoporous LTO hollow spheres present a remarkable high rate capability and stable long‐term cycling capacity,^27^ while above structures are easily destroyed during application in full LTO‐based power battery. In addition, a disadvantage of the porous LTO hollow spheres is that they are generally prepared by spray‐drying and hydrothermal processes, which require complex multi‐step processes that consume a large amount of energy.^22^, ^23^

Although many microsized LTO spheres were prepared, the tap density is still relatively lower due to their highly porous structure. Microsized LTO assembled from nanosized primary particles with a compact micrometer structure can match the high tap density requirement, potentially providing excellent cycling and high rate performance as required for grid energy storage and electric vehicles (EVs).^5^, ^34^ The introduction of micropores in microsized LTO having a compact structure may not compromise the tap density and at the same time possess excellent rate and cycling performance of LTO. This motivates the development of a method to prepare microsized LTO with a compact structure in combination with the presence of micropores. Recently, it was reported that ammonium bicarbonate (NH_4_HCO_3_) as an electrode template can enhance the interconnectivity of the available porosity in Li‐ion electrodes to improve the rate performance.^31^, ^37^ However, the uniform distribution of NH_4_HCO_3_ in the electrode matrix is difficult to achieve during preparation of electrodes by adding the NH_4_HCO_3_ to slurry the electrode. In addition, very long time is needed to dry the LTO electrode with NH_4_HCO_3_ and decompose NH_4_HCO_3_ to form pores.

In this work, instead of addition of the NH_4_HCO_3_ into the electrode slurry, the NH_4_HCO_3_ was added to the precursors of LTO as a template to synthesize the LTO microbars with micropores. The process is very simple because creating micropores in LTO microbars can be achieved together with the preparation of LTO. The subsequent in situ growth of the spinel LTO nanocrystals result in both high tap densities (1.20 g cm^−3^) and an excellent high rate performance. The as‐prepared LTO microbars also have a very small specific area (6.11 m^2^ g^−1^) combined with a large ionic conductivity (5.53 × 10^−12^ cm^−2^ s^−1^). The specific charge capacity of the LTO microbars at 1, 10, 20, and 30 C results in 161.5, 141.0, 132.1, and 129.3 mAh g^−1^, respectively. Only 6.0% capacity loss at a high rate of 10 C is found after 500 and 1000 cycles, the charge capacity still remains 83.3%. In situ growth of the rich micropores in microsized LTO using NH_4_HCO_3_ can not only provide a high tap density of LTO to enhance the volumetric energy density, but also provides abundant Li‐ion transport channels responsible for a high rate charge and discharge. This method provides an effective and universal method to create micropores in the microsized electrode materials for achieving high rate performances and long‐term cyclic and high volumetric energy densities.

## 2 Results and Discussion

**Figure 1:**
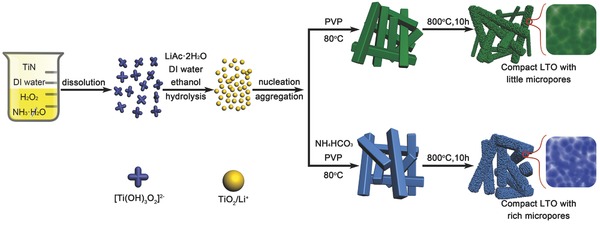
Schematic illustration of synthesis of LTO with and without NH_4_HCO_3_.

The synthetic procedure to obtain the LTO microbars, with and without using NH_4_HCO_3_, is summarized in **Figure** **1**. First, the titanium nitride (TiN) nanopowders were dissolved in deionized water with assistance of hydrogen peroxide (H_2_O_2_) and ammonia solution (NH_3_·H_2_O). The color of the solution gradually changed from a black to dark‐green, and finally to a transparent yellow solution to form the peroxo‐titanium complex ([Ti(OH)_3_O_2_]^−^). After addition of deionized water and ethanol, the yellowish peroxo‐titanium complex solution gradually becomes milk‐like due to formation of the TiO_2_/Li^+^ nanoparticles. After the addition of lithium acetate dihydrate (LiAc·2H_2_O) and polyvinyl pyrrolidone (PVP), the nanoparticles were assembled to form the TiO_2_/Li^+^ microbars. The formation of microbars may be derived from the nucleation and growth of the tiny amorphous TiO_2_/Li^+^ particles induced by PVP. As illustrated in our previous work,^6^ uniform monodisperse TiO_2_/Li^+^ nanospheres were formed in a stable alkaline environment using lithium hydroxide monohydrate (LiOH·H_2_O), while the addition of single neutral LiAc·2H_2_O led to the formation of TiO_2_/Li^+^ microspheres. The pH value of the precursor solution governs the morphology of the TiO_2_/Li^+^. When the PVP and LiAc·2H_2_O were used simultaneously, the PVP acted as a structure‐directing agent that facilitated the formation of the microbars by directed assembling of the TiO_2_/Li^+^ nanoparticles. The TiO_2_/Li^+^ microbars were annealed at 800 °C to obtain the LTO microbars where NH_4_HCO_3_ was used to regulate the porous structure of LTO.

The X‐ray diffraction (XRD) analysis was carried out to identify the crystallographic structure of the LTO microbars prepared at 800 °C. The reflections of both the LTO‐P and LTO‐P‐N patterns are well defined and consistent with the JCPDS Card No. 49‐0207 and can be indexed to the spinel structure of LTO with the space group Fd3m, indicating that pure and highly crystalline LTO is formed. The addition of NH_4_HCO_3_ almost does not influence the crystalline formation of LTO. However, samples are annealed at lower temperatures, such as 600 and 700 °C, the XRD patterns demonstrate the presence of impurity phases, including anatase TiO_2_ obviously appeared (Figure S2, Supporting Information). The peak widths at 600 and 700 °C are significantly wider compared to that at 800 °C. The presence of impurity phases at lower annealing temperatures indicates that the TiO_2_/Li^+^ need to be annealed at least at 800 °C in order to obtain pure LTO microbars.

**Figure 2:**
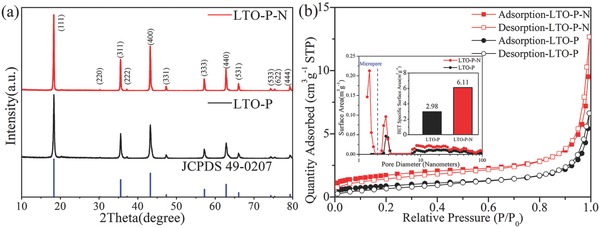
a) X‐ray diffraction (XRD) patterns and b) nitrogen adsorption–desorption isotherms of LTO‐P and LTO‐P‐N, the inset presents the pore size distribution of the LTO‐P and LTO‐P‐N microbars based on the non‐local density functional theory (NLDFT) method.

**Figure** **2**b shows the nitrogen adsorption/desorption isotherms of the LTO microbars. It is observed that the nitrogen adsorption/desorption isotherms of LTO‐P and the LTO‐P‐N are of type IV, indicating that these materials maintain the mesoporous structure, however the pore sizes of LTO‐P and the LTO‐P‐N are quite different. The addition of NH_4_HCO_3_ during the synthesis of LTO increases the micropores content in LTO, aiming at improvement of the Li‐ion diffusion to improve the charge and discharge capacities at high rates. Brunauer–Emmette–Teller (BET) specific surface area and its corresponding pore volumes of LTO‐P are 2.98 m^2^ g^−1^ and 0.0101 cm^3^ g^−1^, respectively. These very small BET surface area and pore volumes illustrate very dense structures with nearly no pores inside the LTO microbars resulting in high tap density. Adding NH_4_HCO_3_ to the synthesis of LTO results in an increase of the BET surface area and pore volumes to 6.11 m^2^ g^−1^ and 0.0188 cm^3^ g^−1^, respectively. The BET specific surface area contribution of the micropores is as high as 2.78 m^2^ g^−1^. The LTO‐P and LTO‐P‐N microbars possess a high tap density of 1.27 and 1.20 g cm^−3^, respectively, which demonstrates that the created micropores in the LTO microbars by the NH_4_HCO_3_ leads only to a small decrease in tap density.

**Figure 3:**
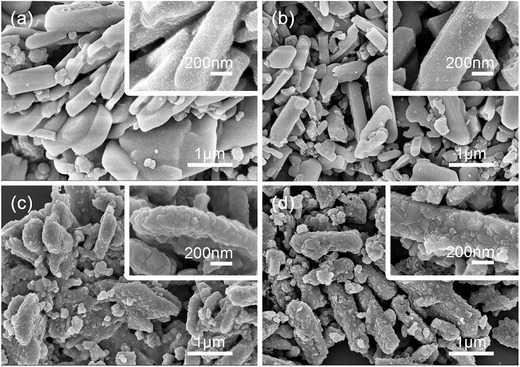
SEM images of precursors of a,b) LTO‐P and LTO‐P‐N microbars and c,d) LTO‐P and LTO‐P‐N microbars annealed at 800 °C.

The morphologies and structures of the LTO‐P and the LTO‐P‐N before and after annealing were examined by scanning electron microscopy (SEM) and high‐resolution transmission electron microscopy (HRTEM). As shown in **Figure** **3**a,c the TiO_2_/Li^+^ particles with and without NH_4_HCO_3_ are rectangular shaped microbars with a length and width of about 2 µm and 200–300 nm, respectively. The microbars obtained after annealing consist of densely packed primary LTO nanocrystals and the size of the LTO‐P‐N particles is larger than the LTO‐P particles (Figure 3c,d). According to the Debye–Scherrer equation, the average diameter of LTO‐P and LTO‐P‐N primary nanocrystals from XRD are 30.4 and 46.6 nm, respectively, suggesting that the addition of NH_4_HCO_3_ facilitates the growth of the primary crystallites.

**Figure 4:**
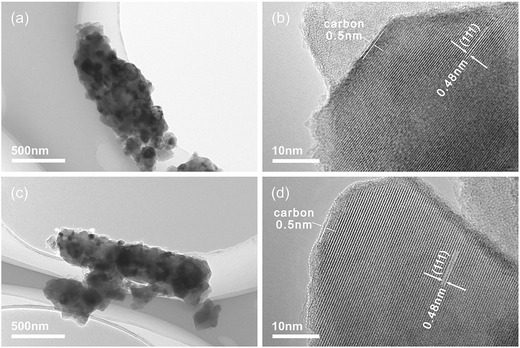
a,b) HRTEM images of LTO‐P microbars prepared at 800 °C and c,d) HRTEM images of LTO‐P‐N microbars prepared at 800 °C.

TEM images show that the microbars prepared at 800 °C are not hollow, but have a solid structure (**Figure** **4**). Both LTO‐P and LTO‐P‐N microbars are in situ grown by spinel LTO nanocrystals to form compact spinel LTO structures and the LTO nanocrystals may benefit for the enhancement of the rate performance of LTO. As shown in Figure 4c,d, the polycrystalline LTO has a well‐defined crystalline structure and the lattice plane spacing of 0.48 nm is consistent with the (111) facet of spinel LTO. The LTO nanocrystals are coated by a carbon film of ≈0.5 nm thick, which is derived from the decomposition of PVP. The carbon layer offers an electronic conducting network to facilitate the electron transport. The electronic conductivity of LTO‐P and LTO‐P‐N microbars is measured to be 3.4 × 10^−6^ and 3.6 × 10^−6^ S cm^−1^, respectively, which is much higher than the intrinsic electronic conductivity of LTO (10^−13^ S cm^−1^). This result presents that the LTO‐P and LTO‐P‐N microbars have similar electronic conductivity, indicating that the different rate performance of LTO‐P and LTO‐P‐N microbars is mainly attributed to their different ionic conductivity. According to the thermogravimetric analysis (TGA) curve and Raman spectrum (Figure S3, Supporting Information), the coated carbon is in the amorphous state and the carbon content in the LTO‐P and LTO‐P‐N microbars is ≈2.00 and 1.56 wt%, respectively.

**Figure 5:**
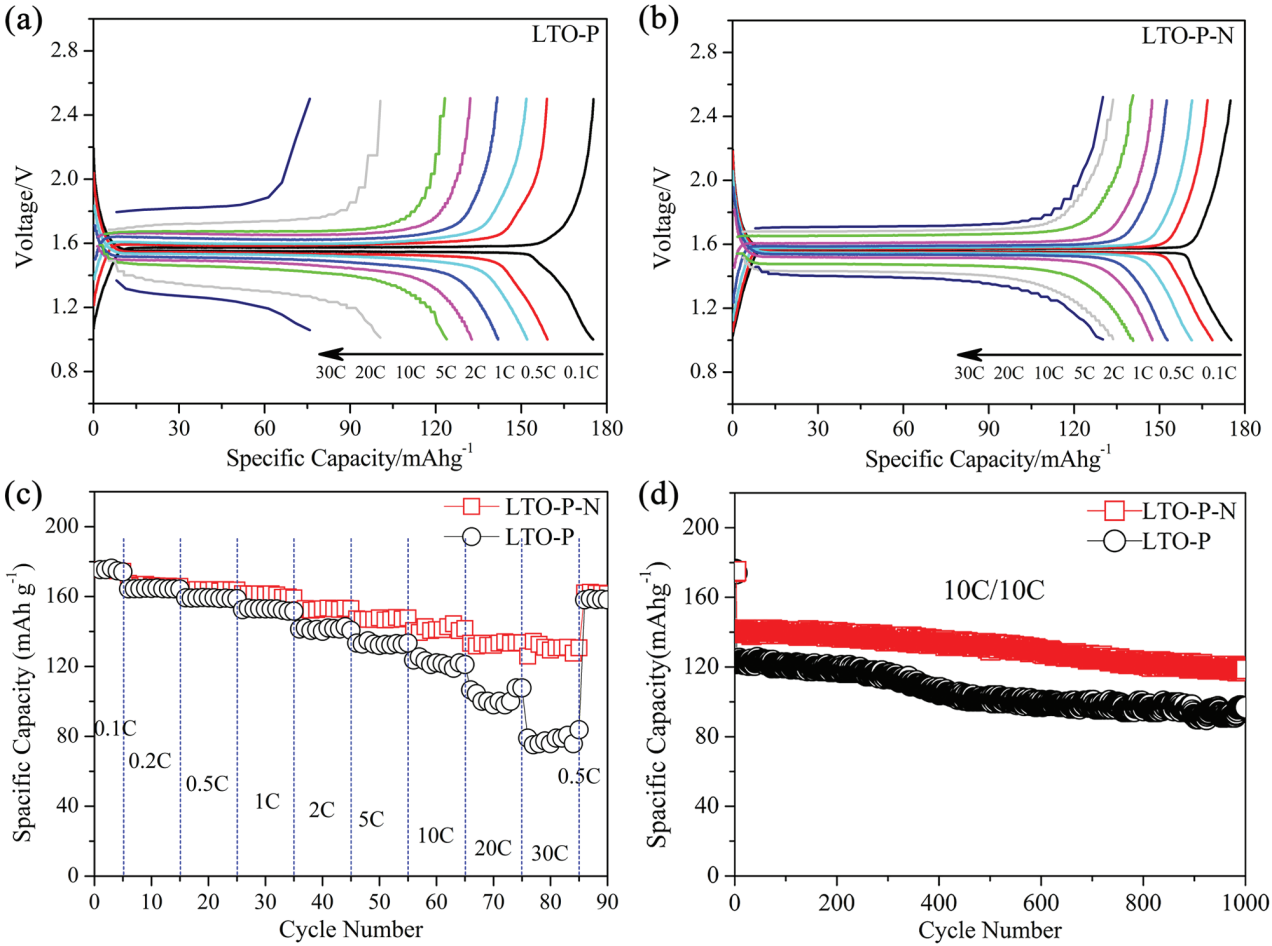
Electrochemical properties of LTO‐P and LTO‐P‐N microbars annealed at 800 °C: a,b) (dis)charge curves, c) specific capacities at different C rates, and d) cycling performance at a rate of 10 C.

In order to identify the effects of the NH_4_HCO_3_ induced micropores on the lithium ion diffusion of LTO‐P‐N, the rate capability of the LTO‐P and LTO‐P‐N samples was examined. It can be seen from **Figure** **5** that at a low C‐rate of 0.1 C, the LTO‐P and LTO‐P‐N electrodes deliver a specific capacity of 175.3 and 175.4 mAh g^−1^, respectively, which is very close to the theoretical capacity of 175 mAh g^−1^. As the current rate increased from 1 to 5, 10 C, the charge specific capacities of LTO‐P‐N are 161.5, 147.7, and 141.0 mAh g^−1^, corresponding to 91.4%, 84.0%, and 80.0% of that at 0.1 C, respectively. Even at very high rates, such as 20 and 30 C, the specific capacities remain as high as 132.1 and 129.3 mAh g^−1^, corresponding to 75.4% and 73.9% of that at 0.1 C, respectively. The specific capacity and capacity retention are among the highest values ever reported for LTO spheres with loosely packed primary particles (Table S1, Supporting Information). It is also worth noting that the (dis)charge overpotentials for the LTO‐P‐N at 10 and 30 C are 0.204 and 0.321 V, respectively, very similar to that of LTO nanosheets.^22^ This small polarization demonstrates that LTO‐P‐N microbars have a small internal resistance for electron and Li‐ion transport responsible for the excellent lithiation and delithiation behavior during the high rate cycling.

However, the charge specific capacity of LTO‐P decreases sharply. As the current rate increased from 1, 5, 10, 20 to 30 C, the corresponding charge specific capacity decreases from 152.9, 132.3, 121.9, 100.0 to 78.1 mAh g^−1^, respectively, which corresponds to 87.3%, 75.6%, 69.6%, 57.1%, and 44.6% of that at 0.1 C. The (dis)charge overpotentials for the LTO‐P at 10 and 30 C are 0.265 and 0.565 V, respectively, which is significantly larger compared to LTO‐P‐N. This result indicates the presence of large polarization in the LTO‐P electrodes, which must be either due to electron or Li‐ion transport. It is well‐known that the LTO materials with small particle size should have fast kinetic behavior. The rate performance of LTO‐P‐N (46.6 nm) and LTO‐P (30.4 nm) seems contradictory with above viewpoint. Because the LTO‐P‐N and LTO‐P have similar electronic conductivity, the much better rate performance of LTO‐P‐N than LTO‐P indicates that LTO‐P‐N has a higher Li‐ion conductivity. The LTO‐P‐N microbars possess richer micropores created by NH_4_HCO_3_ than that of LTO‐P in situ grown by spinel LTO nanocrystals with very dense structure and nearly no pores. The richer micropores in the LTO‐P‐N materials enable more facile Li‐ion transportation, resulting in much better kinetic behavior. Figure 4d demonstrates that the LTO‐P‐N microbars also possess superior cyclic stability with only 6.0% capacity loss at a high rate of 10 C after 500 cycles and 17.7% after 1000 cycles. In sharp contrast, the capacity loss of LTO‐P samples after 500 and 1000 cycles at the same conditions amounts 15.6% and 21.1%, respectively. The stable cycling properties of LTO‐P‐N microbars at high rate (dis)charge cycling can be attributed to lower polarization induced by the micropores.

**Figure 6:**
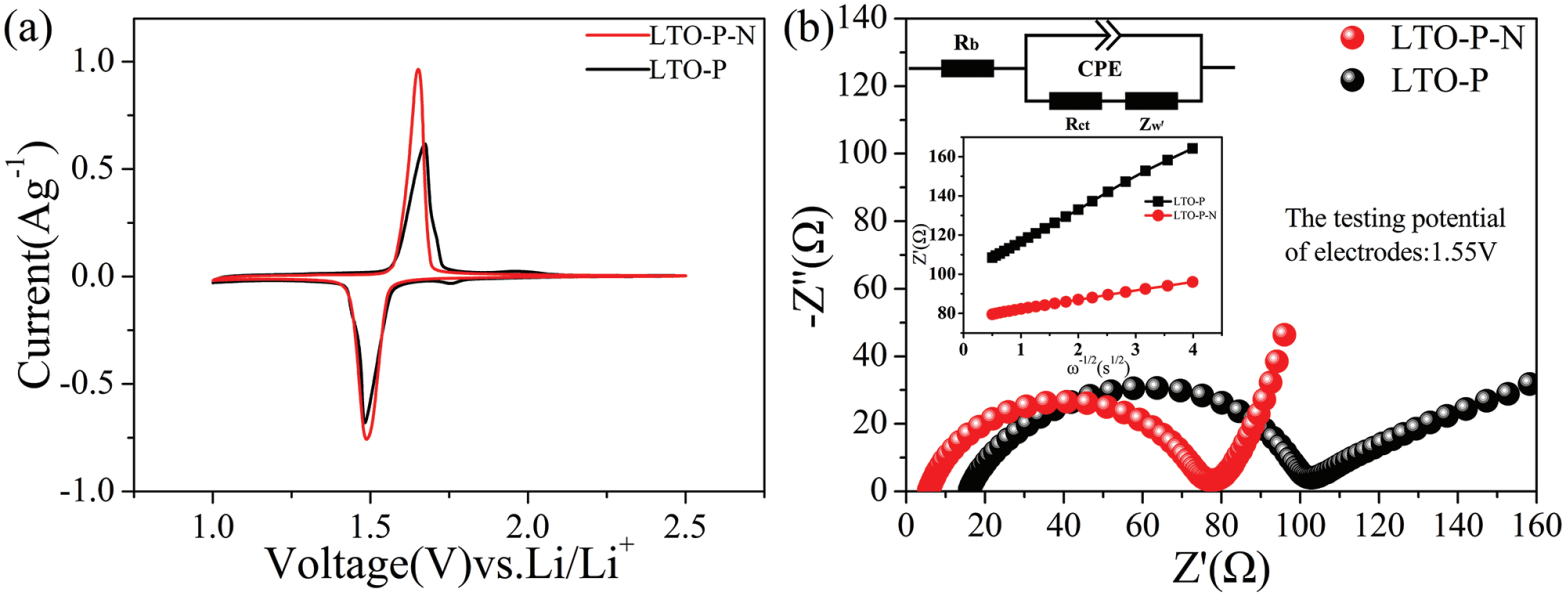
a) CV and b) EIS curves of LTO‐P and LTO‐P‐N microbars annealed at 800 °C.

Cyclic voltammetry (CV) was carried out to investigate the kinetic behavior of LTO‐P and LTO‐P‐N (**Figure** **6**a). The oxidation peak and reduction peak of LTO‐P‐N are located at 1.652 and 1.488 V, corresponding to the LTO Li‐ion extraction and insertion. However, the oxidation peak and reduction peak of LTO‐P are located at 1.673 and 1.483 V. Therefore, the voltage gap of oxidation peak and reduction peak for LTO‐P‐N (0.164 V) is smaller than that of LTO‐P (0.190 V). In addition, the peak current density of LTO‐P‐N is much larger than that of LTO‐P. All these results reflect the excellent kinetic properties of the LTO‐P‐N compared to the LTO‐P materials, induced by the presence of the micropores in the LTO‐P‐N materials. The electrochemical properties of the LTO microbars were further demonstrated by electrochemical impedance spectroscopy (EIS) measurement, fitted by an equivalent circuit model using the Z‐view software shown in Figure 6b. As shown in Figure 6b, the real axis refers to the Ohmic resistance (*R*_b_). The depressed semicircle from high to medium frequencies is attributed to the charge‐transfer resistance (*R*_ct_) and constant phase elements (CPE) of the electrode. The slope line at low frequencies corresponds to the Warburg impedance (*Z*_w_), which is related to the Li‐ion diffusion in the nanospheres.^6^, ^38^, ^39^ The simulation results and the calculated Li‐ion diffusion coefficients (*D*_Li_) are shown in Table S2 (Supporting Information). The *R*_b_ of LTO‐P and LTO‐P‐N microbars are 15.81 and 5.684 Ω and the *R*_ct_ of LTO‐P and LTO‐P‐N are 79.0 and 68.6 Ω, respectively. The *D*_Li_ for LTO‐P‐N was 5.53 × 10^−12^ cm^2^ s^−1^, which is six times larger than that of LTO‐P (≈9.09 × 10^−13^ cm^2^ s^−1^). The larger *D*_Li_ of LTO‐P‐N provides a quantitative proof that the microspores in the LTO‐P‐N microbars greatly improved the Li‐ion diffusion and transport. Thus, the NH_4_HCO_3_ induced creation of the microporous structure in microsized LTO materials is a facile and cheap method to improve their rate and cycling performance.

## 3 Conclusions

A novel LTO material is prepared using NH_4_HCO_3_ as a template resulting in compact microbars with abundant micropores. The LTO microbars are in situ grown by spinel LTO nanocrystals, which combine both a high tap density (1.20 g cm^−3^) and a small specific surface area (6.11 m^2^ g^−1^) as required for high practical energy densities and a long cycle life. As a result of the micropores and a thin carbon coating layer on the LTO nanocrystals, the LTO microbars have both high electronic conductivity (3.6 × 10^−7^ S cm^−1^) and a large Li‐ion diffusion coefficient (5.53 × 10^−12^ cm^−2^ s^−1^), which are responsible for the exceptionally stable long‐term high rate cyclic performance and high (dis)charge rate properties. The capacity retention after 500 and 1000 cycles at 10 C reached 94.0% and 83.3%, respectively. The specific capacities at 10 and 30C are as high as 141.0 and 129.3 mAh g^−1^. This work demonstrates that in situ addition of a cheap template such as NH_4_HCO_3_ to the LTO precursors is an effective way to create micropores in microsized LTO, which results in a material that combines a long cycle life with excellent rate capabilities and a high volumetric energy density.

## 4 Experimental Section

*Synthesis of the LTO Microbars*: LTO microbars were prepared as follows. First, 0.5 g of TiN nanopowders were scattered in 28 mL deionized water. Subsequently, 16 mL of H_2_O_2_ (30%) and 6 mL NH_3_·H_2_O (25%–28%) were added into the above mixture. And then the mixture was magnetically stirred for 0.5 h at room temperature to form the peroxo‐titanium complex. 50 mL of deionized water and 100 mL of ethanol were then added to the precursor solution under constant magnetic stirring for the hydrolysis of peroxo‐titanium complex. Subsequently, 0.724 g of LiAc·2H_2_O and 0.300 g of PVP were added to the precursor solution, and then 0.148 g NH_4_HCO_3_ was added to the above mixture. After a few minutes stirring, the solution was dried at 80 °C for 48 h, yielding the white powder of amorphous TiO_2_/Li^+^ microbars. Finally, this white powder was further calcined at 600, 700, and 800 °C for 10 h under Ar to form the final LTO black powder (denoted as LTO‐P‐N). For comparison, the LTO without NH_4_HCO_3_ was also prepared using same conditions (denoted as LTO‐P).

*Materials Characterization*: The LTO microbars compositions were characterized by XRD (Rigaku D/max 2500/PC using Cu Kα radiation with λ = 1.5418 Å). X‐ray profiles were recorded between 10° and 80° (2θ). The morphologies and microstructures were performed on field emission scanning electron microscope (FESEM, ZEISS SUPRA) at 5 kV and HRTEM (TECNAIG2 F30) at an accelerating voltage of 300 kV. The Raman spectra were carried out on a Raman Spectrometer (HORIBA Labram HR Evolution) with a 532 nm Ar‐ion laser. The carbon content in Li_4_Ti_5_O_12_ microbars was determined by a NETZSCH STA449F3 thermal analyser under an air flow rate of 10 °C min^−1^ from room temperature to 800 °C. The nitrogen adsorption/desorption isotherms were obtained at 77 K by using an automated adsorption apparatus (Micromeritics ASAP 2020). The surface area was calculated based on the BET equation and pore size distribution was calculated from density functional theory (DFT) methods. The electronic conductivities of microporous Li_4_Ti_5_O_12_ microbars were measured using the four‐point probe method. The LTO materials were made into a small disk (thickness: ≈2 mm, diameter: ≈20 mm) by a tablet machine with special models. The electronic conductivities of microporous Li_4_Ti_5_O_12_ microbars were measured by the four‐point probe method using four‐point probe equipment (LORESTA GP, MCPT610).

*Electrochemical Characterization*: Electrochemical performance tests of the LTO microbars electrodes were performed using CR2032‐type coin half‐cells, which were assembled in an argon‐filled glove box with O_2_ and H_2_O contents less than 0.1 ppm. The LTO electrodes were prepared as follows: 80 wt% as‐prepared materials, 10 wt% conductive Super P, and 10 wt% polyvinylidene fluoride (PVDF) were homogeneously mixed in an N‐methylpyrrolidone (NMP) solvent to obtain a slurry, which was uniformly coated onto a copper foil current collector (the LTO electrode loading: ≈2.0 mg cm^−2^ for the tested samples in half cells). The as‐prepared electrode was dried 110 °C for 24 h in a vacuum drying oven. The metallic lithium was used as the anode. A 1 m LiPF_6_ solution in ethylene carbonate (EC)/diethyl carbonate (DEC)/ethyl methyl carbonate (EMC) (volume ratio: 1:1:1) was used as the electrolyte. Microporous polyethylene (Celgard 2500) served as the separator. The assembled half cells were galvanostatically cycled between 1.0 and 2.5 V (on a Land 2001A battery testing system) at different current densities (the current density of 1 C corresponds to 175 mA g^−1^) at room temperature. EIS was carried out at the half discharge state of the LTO electrode by a VMP3 multichannel electrochemical station and applied a perturbation voltage of 5 mV in the frequency range from 100 kHz to 100 mHz. CVs were recorded using the same electrochemical workstation (Solartron Analytical 1470E cell Test System) at a scan rate of 0.1 mV s^−1^ in the range of 1.0–2.5 V.

## Supporting information

As a service to our authors and readers, this journal provides supporting information supplied by the authors. Such materials are peer reviewed and may be re‐organized for online delivery, but are not copy‐edited or typeset. Technical support issues arising from supporting information (other than missing files) should be addressed to the authors.

Supplementary
