# Supplementary material for: High‐Density Microporous Li4Ti5O12 Microbars with Superior Rate Performance for Lithium‐Ion Batteries
Source: Adv Sci (Weinh). 2017 Jan 25;4(5):1600311. doi: 10.1002/advs.201600311 (PMC5441411; doi:10.1002/advs.201600311)
Supplement: Supplementary file 1 — Supplementary [file ADVS-4-na-s001.pdf]

Copyright WILEY-VCH Verlag GmbH & Co. KGaA, 69469 Weinheim, Germany, 2017.

ADVANCED SCIENCE
Open Access

Supporting Information

for *Adv. Sci.,* DOI: 10.1002/advs.201600311

High-Density Microporous $Li_4Ti_5O_{12}$ Microbars with Superior Rate Performance for Lithium-Ion Batteries

Linkai Tang, Yan-Bing He, Chao Wang, Shuan Wang, Marnix Wagemaker, Baohua Li, Quan-Hong Yang, and Feiyu Kang*

Supporting Information

High-density microporous $Li_4Ti_5O_{12}$ microbars with superior rate performance for lithium ion batteries

By Linkai Tang,Yan-Bing He, Chao Wang, Shuan Wang, Marnix Wagemaker, Baohua Li, Quan-Hong Yang, Feiyu Kang*

L. Tang, Prof. Y. -B. He, C. Wang, S. Wang, Prof. B. Li, Prof. Q. -H. Yang, Prof. F. Kang
Engineering Laboratory for the Next Generation Power and Energy Storage Batteries
Graduate School at Shenzhen
Tsinghua University
Shenzhen, 518055, (P. R. China)
E-mail: he.yanbing@sz.tsinghua.edu.cn

Prof. M. Wagemaker
Department of Radiation Science and Technology, Delft University of Technology, Mekelweg 15, 2629JB, Delft, The Netherlands.

L. Tang, C. Wang, Prof. F. Kang
Laboratory of Advanced Materials, Department of Materials Science and Engineering, Tsinghua University, Beijing 100084, PR China.

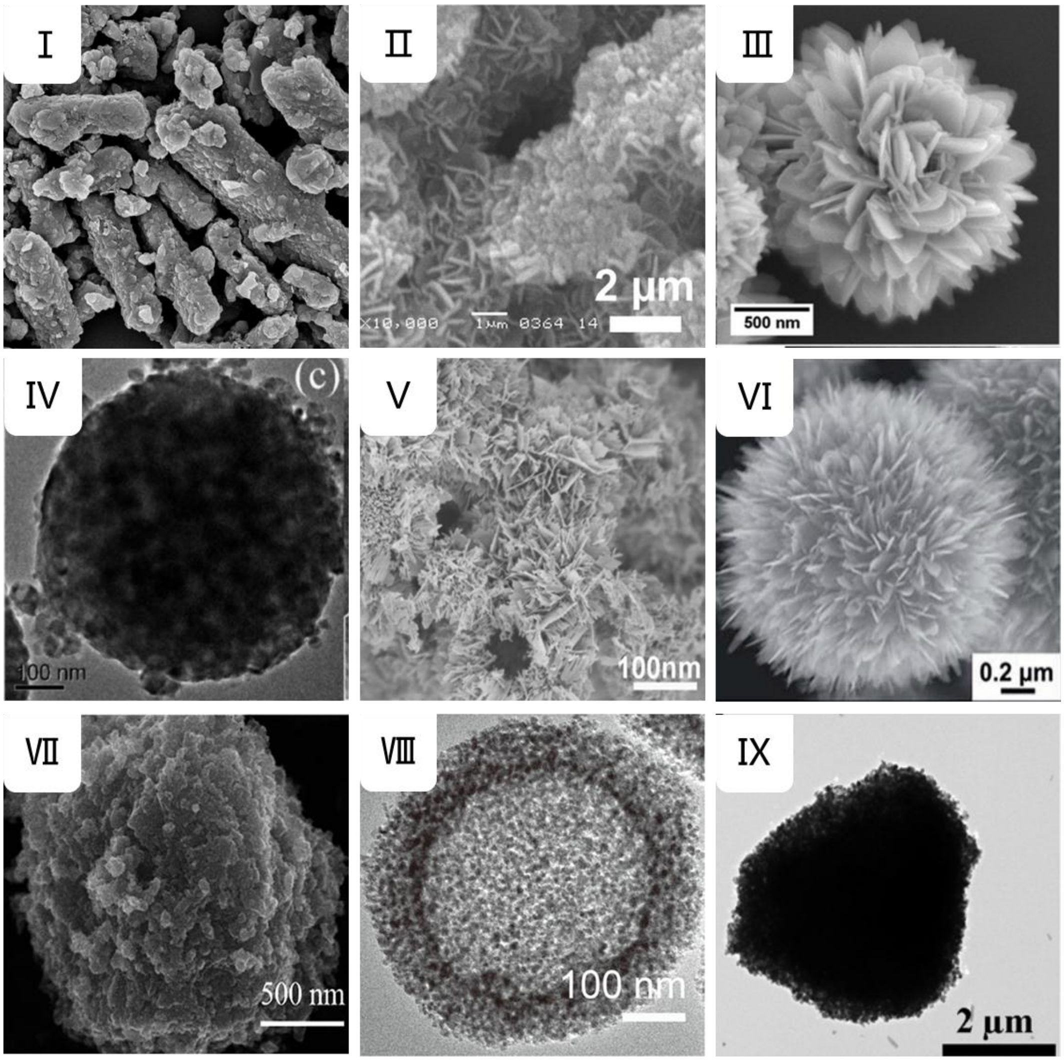

Figure S1. The morphologies and electrochemical performance of the reported references for microsized LTO materials: (I) this work; and (II~IX) reference 1~8.

Table S1. The electrochemical performance of the microsized LTO materials reported references in **Figure S1**.

Sample number	Morphology	Size (μm)	Tap density	Specific surface area (m^2 g^{-1})	Rate performance (mAh g^{-1})	Cycle rate and capacity retention after cycles
Ⅰ	This work Microbars	2	1.20	6.11	10C:140.1	10 C, 500 cycles, 94% retention
Ⅱ	Hierarchically Porous[1]	2	no mention	30	10C: 155<br>30C: 123	5C, 500 cycles,<br>98% retention
Ⅲ	Flower like[2]	0.9±0.1	no	44.0	10C: 141	20C, 300 cycles, 87%

			mention		50C: 123	retention
Ⅳ	submicrospheres [3]	0.72±0.03	1.62	40.2	10C: 115	10 C, 100 cycles, 86.1% retention
Ⅴ	urchin-like microspheres[4]	0.5	no mention	140	10C: 134.6<br>20C: 120	2C,100 cycles 98.1% retention
Ⅵ	dandelion-like microspheres[5]	1	no mention	no mention	10C: 116.9	0.69C, 100 cycles 84.8% retention
Ⅶ	Nano/micro structured porous[6]	1–4	no mention	80.1	10C: 144<br>20C: 132	5C, 100cycles 97.3% retention
Ⅷ	Hollow Spheres[7]	0.4	no mention	220	10C: 115<br>20C: 104	5C, 300 cycles, 88% retention
Ⅸ	hierarchically porous microspheres[8]	4	no mention	57.5	10C: 116<br>20C: 92.3	2 C, 200 cycles 95.2% retention

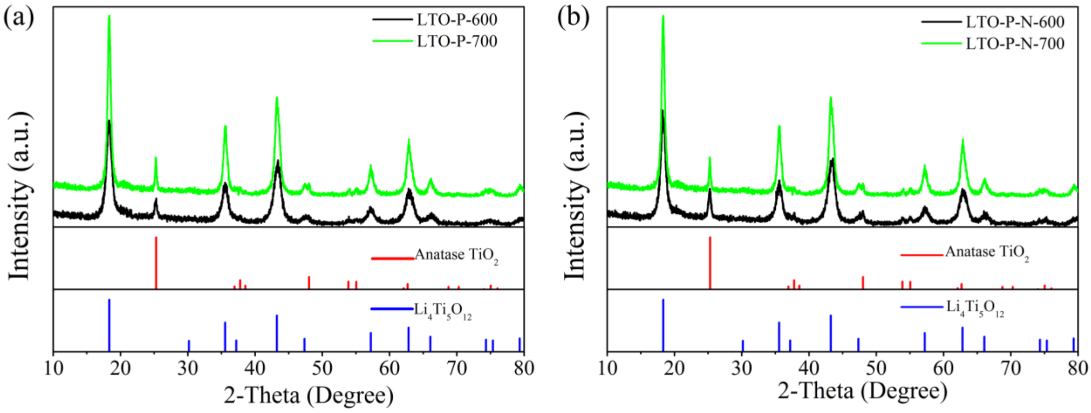

Figure S2. (a) XRD patterns of LTO-P microbars at 600 °C, 700 °C respectively. (b) XRD patterns of LTO-P-N microbars at 600 °C and 700 °C respectively.

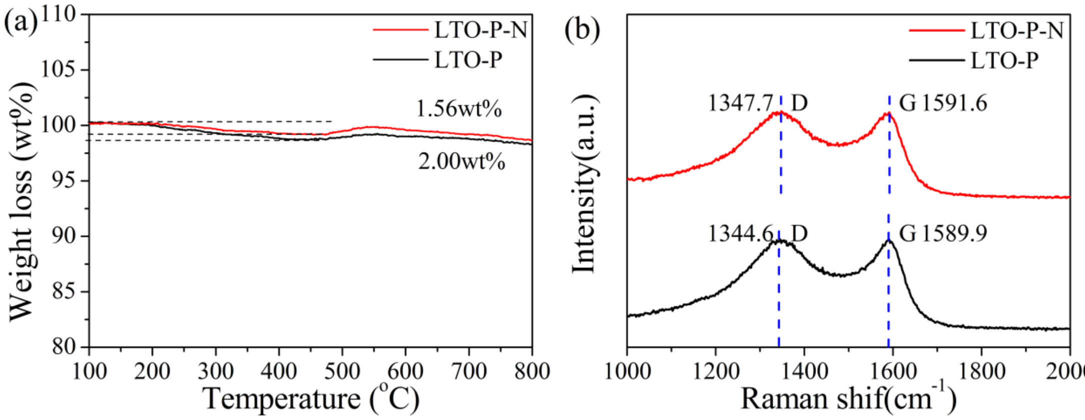

Figure S3. (a) Thermogravimetric analysis, and (b) Raman spectra of LTO-P and LTO-P-N microbars annealed at 800 °C.

Calculation of Li-ion diffusion coefficient (D_{Li})

Electrochemical impedance spectra (EIS) were used to evaluate D_{Li} through the particles. D_{Li} was calculated according to the following equation. [9]

$$D_{Li} = \frac{R^2T^2}{2A^2n^4F^4C^2\sigma^2} \tag{1}$$

where *R* is the gas constant, *T* is the absolute temperature, *A* is the surface area of the electrode, *n* is the number of electrons transferred in the half-reaction for the redox couple, *F* is the Faraday constant, and *C* is the concentration of lithium ions. *C* was 0.014 mol cm^{-3}, which was calculated according to the reference.[10] The lithium ion insertion mechanisms of $Li_{4/3}Ti_{5/3}O_4$ is given by:

$$Li_{4/3}Ti_{5/3}O_4 + Li^+ + e^- \rightarrow Li_{7/3}Ti_{5/3}O_4 \tag{2}$$

Since the EIS was measured at a half discharge state, and thus, $Li_{4/3}Ti_{5/3}O_4$ is half lithiated state. Then, *C* is given:

$$C = \frac{\left(\frac{4}{3} + \frac{1}{2}\right)\rho}{M} \quad (3)$$

where M is the molar mass, M ($Li_{4/3+1/2}Ti_{5/3}O_4$) =156.5 g mol^{-1}; and ρ is the tap density of the electrode, ρ~1.27 g cm^{-3}. Thereby, C=0.014 mol cm^{-3}, and σ is the Warburg factor, which relates to Z' according to Equation (4). σ can be obtained from the slope of the lines between Z' and $\omega^{-1/2}$ as shown in **Figure 6b**.

$$Z' = R_b + R_{sei} + R_{ct} + \sigma\omega^{-1/2} \quad (4)$$

D_{Li} values for LTO-P and LTO-P-N electrodes after 3 cycles at 0.1C were calculated using Equations (1) and (4), and the simulation results of **Figure 6b** are shown in **Table S2**.

Table S2. The simulation results of **Fig. 6b**

Samples	R_b (Ω)	R_{ct} (Ω)	σ	D_{Li} (cm^{2}s^{-1})
LTO-P-N	5.684	68.6	4.72	5.53×10^{-12}
LTO-P	15.81	79.0	16.33	9.09×10^{-13}

References

[1] G. Hasegawa, K. Kanamori, T. Kiyomura, H. Kurata, K. Nakanishi, T. Abe, *Adv. Energy Mater*. **2015**, *5*.

[2] L. Wang, Y. M. Zhang, M. E. Scofield, S. Y. Yue, C. McBean, A. C. Marschilok, K. J. Takeuchi, E. S. Takeuchi, S. S. Wong, *Chemsuschem* **2015**, *8*, 3304.

[3] C. F. Lin, X. Y. Fan, Y. L. Xin, F. Q. Cheng, M. O. Lai, H. H. Zhou, L. Lu, *Nanoscale* **2014**, *6*, 6651.

[4] J. Cheng, R. C. Che, C. Y. Liang, J. W. Liu, M. Wang, J. J. Xu, *Nano Res.* **2014**, *7*, 1043.

[5] D. Z. Kong, W. N. Ren, Y. S. Luo, Y. P. Yang, C. W. Cheng, *J. Mater. Chem. A* **2014**, *2*, 20221.

[6] F. L. Zhang, B. Xu, G. P. Cao, M. Chu, N. Qiao, G. Wei, Y. S. Yang, *Rsc. Adv.* **2014**, *4*, 53981.

[7] L. Yu, H. B. Wu, X. W. Lou, *Adv. Mater.* **2013**, *25*, 2296.

[8] L. F. Shen, C. Z. Yuan, H. J. Luo, X. G. Zhang, K. Xu, Y. Y. Xia, *J. Mater. Chem.* **2010**, *20*, 6998.

[9] S. S. Zhang, K. Xu, T. R. Jow, *Electrochim. Acta* **2004**, *49*, 1057.

[10] X. Y. Wang, H. Hao, J. L. Liu, T. Huang, A. S. Yu, *Electrochim. Acta* **2011**, *56*, 4065.
